# Functional Surface Display of Laccase in a Phenol-Inducible Bacterial Circuit for Bioremediation Purposes

**DOI:** 10.22034/ibj.22.3.202

**Published:** 2018-05

**Authors:** Mehrnoosh Fathi-Roudsari, Mehrdad Behmanesh, Ali-Hatef Salmanian, Majid Sadeghizadeh, Khosro Khajeh

**Affiliations:** 1 National Institute of Genetic Engineering and Biotechnology, Tehran, Iran; 2 Department of Genetics, Faculty of Biological Sciences, Tarbiat Modares University, Tehran, Iran; 3 Department of Biochemistry, Faculty of Biological Sciences, Tarbiat Modares University, Tehran, Iran

**Keywords:** Laccase, Phenol, Bioremediation, AIDA-I

## Abstract

**Background:**

Phenolic compounds, which are produced routinely by industrial and urban activities, possess dangers to live organisms and environment. Laccases are oxidoreductase enzymes with the ability of remediating a wide variety of phenolic compounds to more benign molecules. The purpose of the present research is surface display of a laccase enzyme with adhesin involved in diffuse adhesion (AIDA-I) autotransporter system on the surface of *Escherichia coli* cells for bioremediation of phenolic compounds.

**Methods:**

The expression of laccase was regulated by a phenol-responsive promoter (a σ^54^promoter). The constitutively-expressed CapR transcription activator was able to induce laccase expression in the presence of phenolic compounds.

**Results:**

Western blot analysis showed the expression and correct transfer of the enzyme to the outer membrane of *E. coli* cells in the presence of phenol. Activity assay confirmed the correct folding of the enzyme after translocation through the autotransporter system. HPLC analysis of residual phenol in culture medium showed a significant reduction of phenol concentration in the presence of cells displaying laccase on the surface.

**Conclusion:**

Our findings confirm that autodisplay enables functional surface display of laccase for direct substrate-enzyme availability by overcoming membrane hindrance.

## INTRODUCTION

Human activities have resulted in the release of a plethora of aromatic and organic chemicals into the environment[1]. Phenols and their halogenated derivatives, poly-aromatic hydrocarbons and pesticides, are classes of toxic environmental pollutants that are suspected for carcinogenicity, teratogenicity, and genotoxicity effects[2,3]. Compared to traditional physico-chemical methods, bio-remediation is generally the safest, least disruptive and most cost-effective treatment for the removal of these toxic compounds. Biological tools such as whole microorganisms or isolated enzymes are able to degrade persistent contaminants into non- or less-toxic compounds[4-7].

Laccases are a diverse family of cuproprotein enzymes that oxidize a broad range of aromatic compounds, including phenols, orthodiphenols, p-dihydroxybenzenes, aminophenols, polycyclic aromatic hydrocarbons, and aromatic polyamines[8]. The active site of these enzymes contain a total of four copper ions arranged as a type 1 blue copper site and type 2 and type 3 copper ions that forms a trinuclear cluster for O_2_ binding and activation[9]. Laccases catalyze oxidation of the phenolic compounds with concomitant reduction of oxygen to water[10]. Due to wide reaction capabilities and broad substrate specificity, these enzymes have great biotechnological potentials, including bioremediation, xenobiotic degradation, polymer synthesis, textile dye bleaching, pulp, and paper bleaching as well as biosensor applications[11,12].

Previously, we have prepared a genetically-modified *Escherichia coli* capable of expressing a laccase enzyme in the presence of common phenolic pollutions[13]. A transcription activator (CapR) was constitutively expressed inside the cells but triggered the expression of the laccase gene when exposed to phenolic pollutions[13,14]. It has been shown that upon the presence of phenolic contamination, laccase is able to be expressed and accumulated inside the cells[13]. However, a general problem in using whole microorganisms for bioremediation purposes is bio-availability. Intracellular presence of the enzymes restricts the accessibility towards substrates, therefore decreases the efficiency of bioremediation.

Transfer of the biocatalyst to the cell surface will result in direct contact of the enzyme and substrates without the need for the contaminants to cross membranes[5,15-20]. Among different ways of transferring enzymes to the outer membrane of Gram-negative bacteria, an *E. coli* virulence factor, adhesin involved in diffuse adhesion (AIDA-I), has been widely used. Naturally, the N-terminal signal sequence of AIDA-I is followed by a passenger domain that is transported across the cell envelope through a translocator domain in the C-terminal[21-24]. Here, we have fused the translocator domain of AIDA-I to the C-terminal of the locally-isolated thermostable laccase in our CapR-regulated inducible system. Therefore in the environments with phenolic contaminations, the aromatic molecules that pass through the membrane will result in the expression of chimeric laccase-AIDA gene through activation of CapR. Laccase translocation to the outer cell membrane can increase the exposure of enzyme-substrate and facilitates the detoxification procedure.

Successful display of laccase on the surface of *Bacillus subtilis* spores, *Pseudomonas putida*, and *Saccharomyces cerevisiae* has previously been reported, but there is no report on the surface display of laccase in *E. coli*[8,9,25-27]. Here, we have shown that autodisplay can be used for surface expression of the laccase enzyme, leading to development of a whole cell biocatalyst. Laccase displayed on the surface of the cells showed activity towards its synthetic and natural substrates such as syringaldazine (SGZ) and phenol.

## MATERIALS AND METHODS

### Chemicals and reagents

Taq DNA polymerase, high fidelity PCR enzyme mix, restriction endonucleases, and Ins/TA clone PCR cloning kit were obtained from Fermentas (Germany). SGZ and 3,3’-diaminobenzidine (DAB) were purchased from Sigma (St. Louis, MO, USA). All other chemicals were from Merck (Darmstadt, Germany) with reagent grade. Antibodies were prepared from Biovision (USA).

### DNA manipulation

The previously reported pCap-lac plasmid was purified and digested with *Bam* HI and *Sma* I enzymes[13]. In this construct, a laccase gene, which had formerly been isolated from a local *Bacillus* species (GenBank accession no. FJ663050), was placed under the control of a phenol-inducible Po promoter[13]. Subsequently, AIDA-I transfer unit (TU) region (linker and β-barrel) was amplified using pAng-AIDA plasmid (a generous gift from Dr. Michael Mourez) with forward and reverse primers containing *Bgl* II and *Sma* I sites, respectively. The sequences of the primers are presented below:

AIDAF: AGATCTCTTAATCCTACAAAAGAAAGT

AIDAR: CCCGGGTTAGAAGCTGTATTTTATCCC

The amplified fragment was cloned in pTZ57R/T and digested with the mentioned enzymes. Then the TU was joined in frame with laccase gene in the pCap-lac plasmid. The final construct was named pCaP-lac-TU, and its authenticity was confirmed by sequencing.

### Growth condition and expression of laccase in the presence of inducer

Both pCap-lac and pCap-lac-TU were transformed into chemically prepared *E. coli* BL21 competent cells, and the recombinant clones were selected on Lauria-Bertani (LB) medium containing 100 µg/mL of ampicillin. Five mL of LB medium was inoculated with single colonies of selected transformants and incubated at 37 °C overnight. Subsequently, 50 mL fresh medium was inoculated with the overnight preculture and incubated at 37 °C until an OD_600_ of 0.5 was reached. The induction was carried out with the addition of 100 nm phenol at 37 °C for 4 hours[13].

### Cell fractionation and outer membrane preparation

First, 1 mL of the overnight culture was inoculated into 20 mL fresh medium and grown until reached an OD600 of 0.5-0.6. Then phenol was added, and expression was carried out for four hours. After harvesting and washing with 0.2 M Tris-HCl, pH 8, cells were sonicated for total protein analysis, or differential cell fractionation was performed[28,29]. The protein accessibility test was also carried out in order to find whether laccase is exposed to the surface side of the outer membrane or trapped to the periplasmic side. After expression of laccase, cells were harvested and resuspended in PBS containing proteinase K (50 µg/mL). Treatment was carried out at 37 °C for 1 hour. The reaction was stopped after three times washing of the cells with PBS containing 5% FBS. The fractionation was carried out to prepare the outer membrane of the treated cells[30-33].

### SDS-PAGE and Western blot

Total cell lysate, outer membrane proteins, and the periplasmic/cytoplasmic fractions of the cells were diluted twofold with the SDS-PAGE sample buffer, loaded on 12.5% SDS-PAGE and blotted to PVDF membrane. A monoclonal anti-His-tag was applied in 1% BSA- Tris-buffered saline, 0.1% Tween 20 at 4 °C overnight. A horseradish peroxidase-conjugated goat anti-mouse polyclonal antibody was applied to the membrane with 1:2500 dilution for one hour to develop the signals.

### Whole cell activity assay

Whole cell activity experiment was carried out through the incubation of the whole cells containing pCap-lac or pCap-lac-TU with SGZ, as a spectrophotometric substrate. After the induction of expression with phenol for four hours, cells were harvested and resuspended in PBS at 4 °C overnight. The cells were then harvested and incubated with 0.05 mM SGZ at 37 °C. After 1 hour, cells were sedimented by centrifugation, and the absorbance of the supernatant was measured at 525 nM.

### Effect of laccase on phenol conversion and HPLC analysis

Fresh medium (20 mL) was inoculated with pre-cultured cells, and phenol (100 nM) was added as the inducer after reaching the suitable turbidity of 0.7-0.8. Cells were incubated with phenol for an additional period of 24 hours. Medium without any cells was used as the control in order to find spontaneous conversion of phenol during 24 hours. After this period, cells were harvested, and 800 µl of each medium was used for isolation of organic compounds. Afterwards, 200 µl trichloroacetic acid was mixed with 800 µl medium and stored on ice for 10 minutes. Then the tubes were centrifuged at 12,000 ×g at 4 °C for 15 min. Supernatant was transferred to new tubes and mixed three times with the same volume of trichloromethane. Lower phase containing trichloromethane and all organic extracts of the medium was transferred to new tubes for evaporation of the solvent. The pellet was applied for the HPLC experiment using C18 reverse phase column. Methanol-water with the percentage of 50:50 was used as the mobile phase with the speed of 1 mL/min.

## RESULTS

### Construction of an inducible laccase-displaying circuit in E. coli

**Fig. 1:**
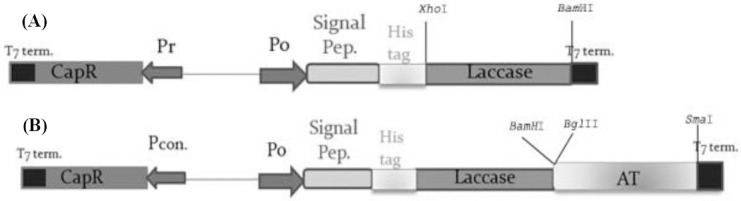
Schematic view of the constructs expressing laccase and laccase-TU fusion protein. (A) The map of the pCap-lac construct. CapR transcription activator is expressed constitutively by the Pr promoter and ends in a T7 terminator. Divergently, Po inducible promoter can be occupied by CapR and results in transcription of the periplasmic signal peptide (pelB), His-tag, and laccase gene in the presence of phenols. The laccase expression is terminated by the T7 terminator. (B) The map of the pCap-lac-TU construct. The TU region corresponding to the linker and β-barrel domain of AIDA-I is joined to the C-terminal of the laccase gene. Upon activation of the Po promoter by CapR, laccase can be expressed in fusion with TU and transferred through the β-barrel to the surface of the outer membrane.

For preparing the pCap-lac-TU construct, the TU of AIDA-I (linker-β-barre) was joined to the C terminal of laccase through compatible ends of *Bam* HI and *Bgl* II. Figure 1 shows the schematic view of the pCap-lac and the pCap-lac-TU final constructs. The recombinant plasmids were transformed into competent *E. coli* BL21 cells and screened on solid media containing 100 µg/ml ampicillin. The reason for choosing *E. coli* BL21, as the host of expression, was the absence of outer membrane protease T (ompT^-^) on the external surface of the bacteria. Since the linker region of the AIDA-I contains a cleavage site for ompT, here, we have used *E. coli* BL21as the host.

### Expression and surface display of laccase

**Fig. 2:**
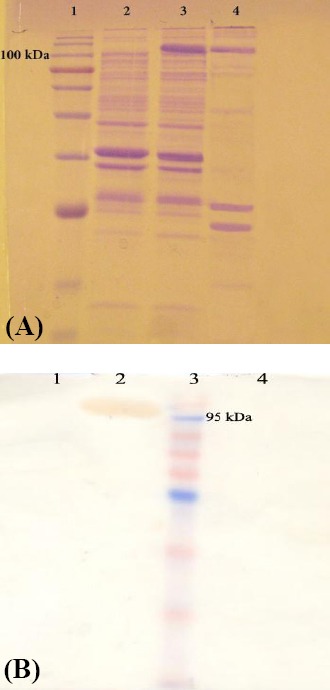
(A) Protein separation using SDS-PAGE analysis. Lane 1, the molecular weight marker; lanes 2 and 3, E. coli cell lysate containing pCap-lac-TU before and after induction with phenol, respectively; lane 4, outer membrane fraction after phenol induction. The chimeric protein with the mass of 106 kDa is obvious after phenol induction. (B) Western blot on cells harboring pCap-lac-TU. The cells were fractioned before and after proteinase K treatment. Lane 1, cytoplasmic/periplasmic soluble proteins; lane 2, outer membrane fraction; lane 3, protein weight marker; lane 4, outer membrane fraction after proteinase K treatment. The band of the fusion protein is present in the outer membrane fraction. After proteinase treatment, the band is disappeared, confirming the correct translocation of the laccase through TU to the outer surface of the cells.

Fusing laccase enzyme (with the molecular mass of 65 kDa) to the TU of AIDA-I results in the formation of a fusion protein with the molecular mass of 106 kDa after the cleavage of signal sequence. Following induction of expression with 100 nM phenol, a band with the molecular mass of 106 kDa was observed in total cell lysate. After cell fractionation, the same band was obvious in the outer membrane fraction (Fig. 2A). Following the induction of expression with 100 nM phenol and cell fractionation, Western-blot analysis showed a band with the correct size, just in the outer membrane fraction of the cells. The band was absent in cytoplasmic/periplasmic fraction, which shows the efficient transfer of the fusion protein to the outer membrane (Fig. 2). Proteinase K treatment was also performed to find the direction of laccase attachment to the outer membrane. Since the proteinase K molecules are unable to pass the outer membrane, degradation of the fusion protein supports the correct transfer of the enzyme to the outer side of the cells. Trapping the laccase in the periplasmic side can protect the fusion protein from degradation. As shown in Figure 2, the band corresponding to the fusion protein was disappeared after proteinase treatment, which proves the surface display of laccase.

It has previously been shown that the formation of disulfide bonds and complete folding of the passenger proteins may result in unsuccessful transfer of the protein through β-barrel to outer membrane[34]. Laccase enzymes contain a disulfide bond between cysteine residues 229 and 322 in crystallographic studies[35]. Although Western-blot analysis of outer membrane fraction with or without proteinase treatment confirmed correct transfer of laccase to the surface, we also prepared the outer membrane fraction from cells, which were grown in highly reducing medium containing 10 mM of 2-mercaptoethanol. Western blot analysis showed no difference in the transfer of fusion protein in normal or reducing conditions (data not shown). It seems that the presence of disulfide bond in laccase structure cannot hinder the transfer of the enzyme through outer membrane.

### Cell surface activity assay of laccase

To investigate the biological activity of the laccase molecules, which were transferred to the outer membrane, whole cells were assayed in the presence of SGZ. After the expression of laccase in the presence of phenol as inducer and dialysis against PBS, cells were incubated with SGZ substrate for one hour. The absorbance of the supernatant at 525 nm showed significantly higher oxidative ability of cells, which harbor laccase-AIDA fusion. Cells containing pCap-lac express laccase inside the cells and show the activity of 0.2 µM/hour in comparison to 2 µM/hour for cells containing pCap-lac-TU. These results prove almost 10 times higher oxidative ability for cells, which display laccase on the surface.

**Fig. 3:**
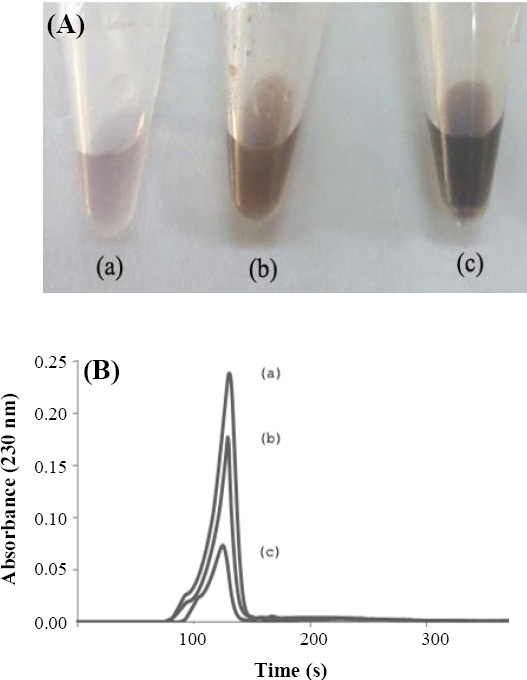
Conversion of phenol and HPLC analysis of residual phenol. (A) The organic fraction of culture medium after 24 hours of phenol (100 nM) treatment. Brown precipitates are the oxidized polymeric forms of phenol remarkably higher in medium of laccase-displaying cells. (B) The supernatant of organic fractions were injected to HPLC. Chromatograms show the amount of residual phenols. (a) Medium without any cells shows a single peak with retention time of 2 minutes. (b) Medium which contained cells with pCap-lac plasmid. (c) Medium contained surface-display laccase cells due to the presence of pCap-lac-TU plasmid. The peak shows an obvious reduction in phenol after treatment with cells displaying laccase.

The biological activity of the displayed laccase was additionally assayed in the presence of phenol as a natural substrate. In the present expression system, phenol not only is an inducer of the laccase expression but also acts as a substrate. After four-hour induction of laccase expression with phenol (100 nM), cells were kept at 37 °C for an additional 24 hours. Medium containing 100 nm phenol was kept at 37 °C for 24 hours, as the control. After harvesting the cells, the organic fractions of the medium were extracted. Figure 3A shows the organic fraction of medium containing cells, which express laccase inside the cells (pCap-lac) or display laccase on the surface (pCap-lac-TU). Obviously, the sample prepared from surface-displayed laccase contained remarkable higher amounts of brown precipitates. These precipitates are known as phenolic polymers formed as the result of oxidation reaction. The organic fraction obtained from pCap-lac containing cells showed much lower amounts of brown precipitates. After centrifugation, the supernatant was analyzed by reverse-phase HPLC to find any residual soluble phenol in the medium. The obtained peaks are shown in Figure 3B. The medium containing 100 nm phenol for 24 hours, without any cells, was used as the control. A single peak with retention time of approximately two minutes was observed. Phenol extracts from medium containing BL21 cells harboring pCap-lac show a reduction in the height of the mentioned peak. Looking at the graph obtained from cells displaying laccase, a remarkable reduction of phenol molecules can be seen. The height and surface area of the peak corresponding to the phenol shows almost four times reduction in comparison to the control.

## DISCUSSION

Laccases (E.C. 1.10.3.2, *p*-benzenediol:oxygen oxidoreductase) are copper-containing oxidoreductase enzymes able to oxidize a variety of aromatic compounds, including mono-, di-, and poly-phenols[36,37]. These enzymes can oxidize, polymerize, degrade, or transform phenolic compounds to less toxic derivatives[38,39]. An interesting characteristic of laccases is their low specificity towards substrates. A diverse and broad range of chemicals, including phenols, dyes, pesticides, endocrine disrupters, and polycyclic aromatic hydrocarbons can be converted by the laccase active site to more benign compounds[40,41]. Therefore, a wide range of applications from food stabilization, dye bleaching, and textile preparation to bioremediation of wastewaters can be catalyzed using these enzymes[4,42].

Although laccases are considered as relatively stable enzymes, prevention of inactivation under industrial conditions still remains a priority. Tolerance of the enzyme towards temperature, pH, organic solvents, and free radicals, which might be produced as the result of laccase function, is a highly important criterion in industrial use of this enzyme. Immobilization by various chemicals or physical methods can increase the stability and tolerance of enzymes to denaturation in harsh industrial situations and extend its functional life-time[5,38,39]. Enzymes can also be immobilized on the surface of live organisms through linking to one of the cell wall or outer membrane molecules. Surface display has other benefits such as direct contact of enzyme with substrates and elimination of the expensive steps of enzyme purification[43].

From different strategies of surface display in Gram-negative *E*. *coli*, we have used the autotransporter system. Functional transport of several enzymes to the surface of *E*. *coli* has been reported frequently[43-45]. Here, we have substituted the passenger domain of

AIDA-I with laccase (laccase-TU) and prepared a pelB signal sequence at the beginning of the coding region. We have also used the inducible pathway of phenol degradation in *Pseudomonas* *putida* KCTC1452; therefore, laccase expression is controlled by the CapR transcription activator and σ^54^-RNA polymerase that recognizes the -12 (GC)/24 (GG) promoter[11].

The present study indicated that in the presence of phenol, laccase was expressed from pCap-lac-TU and successfully transferred to the cell surface. Western blot analyses proved the absence of the chimeric protein in the cytoplasmic fraction of the phenol-treated cells and showed a band with the correct size of chimeric protein in the outer membrane fraction. Proteinase K treatment of whole cells and subsequent disappearing of the laccase-TU band confirmed the correct direction and exposure of the chimeric protein to the outer membrane.

Similar to other immobilization methods, chimerization may also interfere with catalytic properties of the displayed enzymes. Although correct transfer of laccase to the cell surface was confirmed, the functionality of the enzyme was investigated through activity assays. Treatment of the whole cell biocatalysts with SGZ confirmed the remarkable higher oxidative ability of laccase-displaying cells for conversion of this substrate. Additionally, long treatment of cells harboring pCap-lac and pCap-lac-TU with phenol as a natural substrate showed the high oxidative ability of laccase, which was transferred to the surface. Brown polymers, which are the result of phenol polymerization, were remarkably higher in medium containing cells with surface-displayed laccase. The polymerized precipitates were also observed in medium with intracellular-expressing laccase. The negligible amount of phenol poly-merization was also observed in medium without cells, as the negative control, showing the spontaneous polymerization of phenol in the condition of experiment. In agreement to these data, the residual phenol of the culture media was also analyzed using reverse phase HPLC. HPLC analysis of phenol moieties from medium containing BL21 cells with pCap-lac plasmid showed a smaller peak in comparison to the control (medium without cells). It is assumed that phenols can be partially degraded even when laccase is expressed inside the cells. Laccase release, as the result of cell lysis or transfer of a fraction of phenol molecules through the membrane, might be the reason of phenol degradation in this experiment. Presence of the laccase on the surface of *E. coli* cells dramatically reduced the amount of residual phenol in the medium, which indicates the functional transfer of the enzyme and increased bioavailability of substrates.

We conclude that laccase, as the remediating enzyme, is expressed and successfully transferred to the outer surface of *E. coli* in our phenol inducible expression system. The enzyme is capable of passing through the β-barrel of AIDA-I TU. The present study confirms that the displayed enzyme is functional and active on the surface of bacteria and shows the ability to polymerize phenol as the substrate. The cells displaying laccase on the surface can be used as whole-cell biocatalysts.
